# Emerging roles of epigenetic regulation in obesity and metabolic disease

**DOI:** 10.1016/j.jbc.2021.101296

**Published:** 2021-10-09

**Authors:** Yoon Jeong Park, Sang Mun Han, Jin Young Huh, Jae Bum Kim

**Affiliations:** Center for Adipose Tissue Remodeling, Institute of Molecular Biology and Genetics, School of Biological Sciences, Seoul National University, Seoul, South Korea

**Keywords:** obesity, adipose tissue, epigenetics, chromatin remodeling, DNA methylation, three-dimensional chromosome conformation, CCCTC-binding factor, 3C, chromosome conformation capture, DNMT, DNA methyltransferase, FATP1, fatty acid transporter protein 1, LAD, lamin-associated domain, LSD1, lysine-specific demethylase-1, NAD, nucleolus-associated chromatin domain, pCHi-C, promoter-capture Hi-C, SAM, S-adenosylmethione, TAD, topologically associated domain, TET, ten-eleven translocation, TF, transcription factor, WAT, white adipose tissue

## Abstract

Adipose tissue dysfunction is a hallmark of obesity and contributes to obesity-related sequelae such as metabolic complications and insulin resistance. Compelling evidence indicates that adipose-tissue-specific gene expression is influenced by gene interactions with proximal and distal *cis*-regulatory elements; the latter exert regulatory effects *via* three-dimensional (3D) chromosome conformation. Recent advances in determining the regulatory mechanisms reveal that compromised epigenomes are molecularly interlinked to altered *cis*-regulatory element activity and chromosome architecture in the adipose tissue. This review summarizes the roles of epigenomic components, particularly DNA methylation, in transcriptional rewiring in adipose tissue. In addition, we discuss the emerging roles of DNA methylation in the maintenance of 3D chromosome conformation and its pathophysiological significance concerning adipose tissue function.

Obesity, characterized by a multifactorial and chronic condition, is a worldwide epidemic with an estimated prevalence of approximately 1.9 billion adults worldwide in 2016, equating to approximately 39% of adults aged ≥18 years (https://www.who.int/news-room/fact-sheets/detail/obesity-and-overweight). Notably, obesity is a key factor in developing metabolic diseases such as cardiovascular diseases, type 2 diabetes, atherosclerosis, and cancer, which greatly burden individual and public health (1). One hallmark of obesity is the extensive expansion of white adipose tissue (WAT) characterized by maladaptive remodeling events, including increased adipocyte hypertrophy, impaired formation of new adipocytes, and accumulation of proinflammatory immune cells (2). Accordingly, significant advances have been made over the past few decades in understanding obesity-induced aberrant WAT remodeling and its pathophysiology concerning obesity-related metabolic disorders.

WAT is the central controller of lipid and glucose metabolism, which influences systemic energy homeostasis. It actively senses nutritional changes and stores the extra energy as triglycerides or supplies nutrients to other organs (3). In addition, WAT regulates whole-body energy metabolism by communicating locally and with distant tissues through the secretion of various signaling molecules such as adipokines, lipokines, metabolites, and exosomes (3). Multiple factors that are altered in obesity promote aberrant gene expression in WAT, leading to WAT dysfunction (4). Increasing evidence suggests that obesity is closely associated with tissue-specific or even cell-specific epigenome disruption, and aberrant epigenome alteration in WAT may be one of the important mechanisms linking obesity to clinical conditions (5). The study of epigenetics and its involvement in WAT remodeling is still a young research field; however, it now attracts much scientific attention and is growing fast. The development of new experimental techniques for detecting and analyzing epigenetic modifications has contributed to the interest in and advancement of the field. This review summarizes the current understanding of the pathophysiological roles of epigenetics, particularly focusing on DNA methylation in the transcription regulation of WAT function. In addition, the new roles of DNA methylation in maintaining cell-type-specific long-range genomic interactions are discussed.

## 3D chromosomal organization in the regulation of gene expression

**Figure 1:**
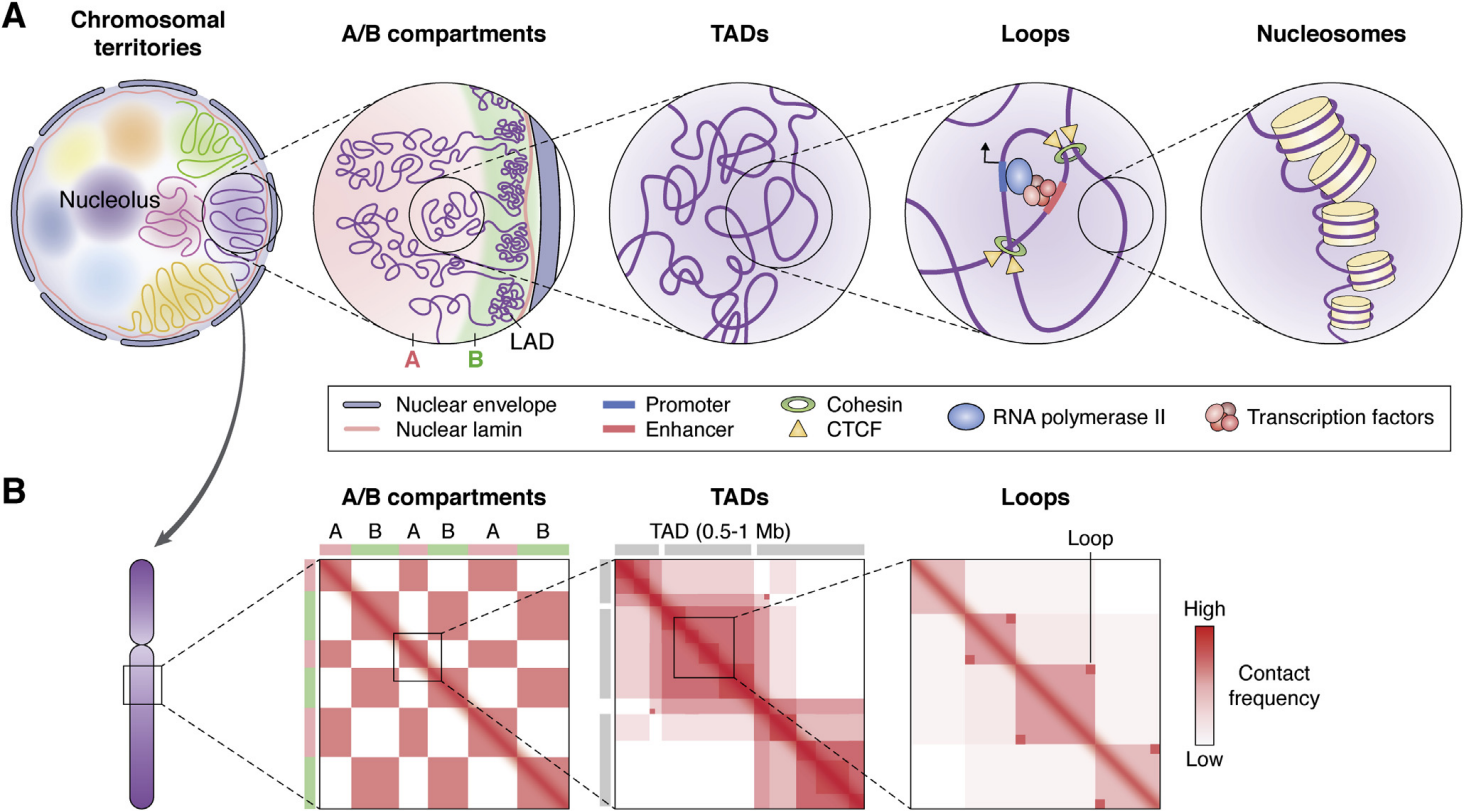
**Chromosomal organization of the eukaryotic genome.***A*, schematic representation of chromosomal organization in the nucleus. Individual chromosomes occupy separate territories in the nucleus. Chromosomes are further divided into A compartments with active chromosome domains and B compartments with inactive chromosome domains such as lamin-associated domain (LAD). Each compartment includes several topologically associated domains (TADs). TADs comprise multiple regulatory loops, which enable a close interaction between distal enhancers and promoters. Chromatin is further divided into histone core and naked DNA. *B*, schematic representation of Hi-C maps at each genomic scale. A and B compartments show a plaid pattern, which is mutually excluded. TAD is defined as DNA sequences exhibiting significantly higher contact frequency with other DNA sequences within the domain than those outside the block, and it ranges from 500 kb to 1 Mb. “Corner-dots” indicate loop structures where both ends are closely associated.

Previously, the early models of transcription often assumed that gene expression is influenced primarily by the linear genome sequence that harbors millions of *cis*-regulatory elements such as enhancers and insulators. However, development of the “Chromosome Conformation Capture” (3C) technology (6, 7) and various genomic approaches based on the 3C technology, such as Hi-C (8), ChIA-PET (9), and capture Hi-C (10), has indicated that chromosomal organization and compaction (Fig. 1*A*) are significant factors for gene expression regulation. At a large scale, individual chromosomes occupy separate territories (11, 12). For instance, large and gene-poor chromosomes are frequently located near the nuclear periphery and form lamin-associated domains (LADs) or nucleolus-associated chromatin domains (NADs). In contrast, small and gene-rich chromosomes tend to be located more at the internal side of the nucleus. Chromosomes are further divided into large chromosome compartments, including A and B compartments. Active and open chromosome domains interact with each other to form A compartments. In contrast, B compartments are formed through the interactions between inactive and closed chromosome domains (8, 13). Importantly, the condensation of large genomes into the 3D space of the nucleus is mediated by the formation of a robust and dynamic looping architecture. These loops exhibit a great variation in length (from a few kilobases [kb] to more than 100 megabases [Mb]) and duration (temporal *versus* persisting loops retaining most part of the cell cycle). Additionally, these spatiotemporal variations in looping structure are suggested to be essential to orchestrate complex regulatory networks and transcription mechanisms. Notably, these looping structures facilitate complex interactions between genomic regions by forming insulated regulatory loop regions, where multiple loops assemble to form topologically associated domains (TADs), ranging from 500 kb to 1 Mb (14, 15). Most of the loops are short-ranged and operate locally within the boundaries of TAD, and these intra-TAD loops impact gene regulation by bringing distant enhancers and promoters together (Fig. 1*B*). Intriguingly, this looping is not limited to simple one-to-one associations and often promotes complex multiway interactions, where enhancers have more than one target gene and a single gene can be regulated by multiple enhancers (16, 17, 18, 19). For instance, a study in which promoter-capture Hi-C was used to identify looping events in 17 human primary hematopoietic cell types (20) revealed approximately 175,000 interactions between promoters and promoter-interacting regions with a median of four interactions per promoter. Furthermore, a study with mouse embryonic stem cells (21) reported that approximately 52% of promoters interact with more than one enhancer. Regarding the enhancers, approximately 70% are linked to 1–5 promoters and 2%–4% to more than five promoters. Thus, these studies suggest that gene expression can be regulated by multiple regulatory elements mediated by a 3D genome architecture. Among the various anchor proteins, TAD maintenance is predominantly mediated by the CCCTC-binding factor (CTCF)/cohesin complex (22, 23). For instance, approximately 15% of CTCF-binding sites coincide with TAD boundaries in pluripotent cells, and a major portion of other CTCF-binding sites is suggested to modulate intra-TAD regulatory looping formation (24). In addition, altered CTCF occupancy has been linked to pathogenicities associated with cancer and genetic diseases through the formation of aberrant chromosome looping between distal *cis*-regulatory elements and their target promoter(s), thus promoting the altered gene expression (25, 26, 27).

## Roles of long-range chromosome interactions in WAT

Recent observations highlight the significance of regulatory circuits mediated by long-range genomic interactions in the regulation of adipocyte gene expression (28, 29). It is now evident that adipocyte chromosome looping is one of the mechanisms that connect a genome-wide association study (GWAS)-identified loci to certain genes related to obesity and metabolic diseases. For instance, Pan *et al.* (28) have demonstrated crucial roles of human adipocyte chromosomal interactions in adipose gene expression and clinical phenotypes associated with obesity (*e.g.*, body mass index [BMI], waist–hip ratio, fasting insulin, and Matsuda index). They performed promoter-capture Hi-C (pCHi-C) in primary human white adipocytes and found that distal elements interacting with promoters through adipocyte chromosomal looping are enriched for adipose-related transcription factor (TF) motifs such as PPARγ and CEBP, contributing to the heritability of *cis*-regulated adipose gene expression. By integrating adipocyte pCHi-C data with GWAS, *cis*-eQTL analyses, and the expression data from GTEX and TwinsUK, they identified four *cis*-eQTL-eGene relationships associated with BMI or obesity-related traits (*e.g.*, rs4776984 and MAP2K5, rs8076131 and ORMDL3, rs1017546 and LACTB, and rs10774569 and ACADS). In another study, Siersbaek *et al.* (29) revealed that the transition from preadipocytes to mature adipocytes is accompanied by the rapid rewiring of chromosome loops linking promoters and enhancers, whereas higher-order chromatin organization at the level of TADs remains relatively constant during adipocyte differentiation. These rapid rewiring events are initiated within 4 h after the induction of adipocyte differentiation; it is tightly coupled to activate poised enhancers (H3K4me1 and H3K4me2) that acquire H3K27ac and the binding of the mediator complex such as MED1, SMC1, and P300. Among 290,000 promoter-anchored chromatin loops, most of these interactions are enriched within TADs, and gene expression is positively associated with the number of promoter-anchored chromatin loops, suggesting the significance of long-range genomic interactions in the robust expression of adipose genes. Notably, key adipogenic TFs and metabolic enzymes (*e.g.*, *Pparg*, *Cebpa*, *Cebpb*, *Fabp4*, *Lpl*, *Pcx*, and *Scd1*) are connected to dynamic super-enhancers; the strength of their promoter-anchored chromatin loops is significantly augmented during adipocyte differentiation.

## Epigenetic modifications of WAT in obesity

### Epigenetics as the bridge between the environment and gene expression

Previously, epigenetics has been defined as heritable changes in gene expression that do not involve alterations in the DNA sequence, and epigenetic patterns are copied through mitotic cell division. However, recent definitions of epigenetics are broader than this, which includes any potentially stable changes in gene expression that occur without altering the DNA sequence. Thus, epigenetic alterations in nondividing cells would not be heritable but may still affect cell-type-specific gene expression and function. Epigenetic modifications include DNA methylation, posttranslational modification of histone proteins, chromatin remodeling, and various RNA-mediated processes. To date, it is known that such epigenetic modifications are molecularly interlinked to altered *cis*-regulatory element activity and chromosome architecture, regulating differentiation, cell-type-specific gene expression, parental imprinting, X chromosome inactivation, and genomic stability. Emerging evidence suggests that epigenetics is a key underlying mechanism linking environmental factors and gene expression in various cell types (30). The activity of many key epigenetic modifiers is influenced by intermediary metabolites, including α-ketoglutarate, S-adenosylmethione (SAM), and nicotinamide adenine dinucleotide, whose abundance is altered in obesity. In particular, the abundance of these metabolites is modulated by microbiota metabolites that act as substrates and cofactors to produce these metabolites. Accordingly, the alteration in microbiota metabolites has been suggested to contribute to abnormal epigenetic regulation in obesity and related metabolic diseases. For instance, global histone acetylation and methylation in multiple tissues are associated with a myriad of metabolites produced by gut microbiota (31). Among various microbiota metabolites, short-chain fatty acids, exclusively produced by microbial fermentation of dietary carbohydrates, inhibit histone deacetylase and, consequently, promote the formation of open chromatin structure (32). In addition, obesity-induced factors provoke the altered expression of key epigenetic modifiers, leading to the perturbation of epigenome landscape, and promote abnormal transcriptional reprogramming (33). In WAT, among various epigenetic modifications, differential variability in histone modifications and DNA methylation is commonly noted owing to epigenetic dysregulation associated with WAT dysfunction, metabolic pathologies, and adverse environment promoted by obesity (Table 1). For instance, clinical studies revealed that obesity-related metabolic parameters such as T2D status and BMI are closely associated with WAT DNA methylation (34, 35, 36, 37, 38). Studies using various mouse models with impaired DNA methylation further indicate the crucial roles of DNA methylation in WAT inflammation, WAT insulin resistance, and browning (39, 40, 41, 42, 43, 44, 45, 46, 47, 48).

**Table 1:** Associated phenotypes of DNA methylation in WAT

Study design and subjects	Associated phenotypes	Reference
Monozygotic twin pairs discordant for T2D	T2D status	(34)
Males and females with a broad range in age	BMI	(35)
Before *versus* after gastric by-pass	Weight loss	(36)
Ex-obese *versus* never-obese	Adipogenesis	(37)
Six-month exercise	Metabolism of adipose tissue	(38)
Treatment with DNMT inhibitor	Fat mass	(68)
Multigenerational high-fat diet intervention	WAT inflammation	(69)
Suppressed DNA demethylation by depleting the adipocyte TET1	Thermogenesis and systemic energy expenditure	(39)
Manipulation of DNA methylation by decreasing the DNMT activity	WAT inflammation and WAT insulin resistance	(40, 41, 42)
Inhibition of DNA demethylation	Adipogenesis	(43, 44)
Inhibition of DNA methylation	Adipogenesis	(45, 46, 47)
Neonatal manipulation of DNA methylation	WAT browning	(48)

### Histone modifications in WAT

Histones undergo various posttranslational modifications, including methylation, acetylation, ubiquitination, and phosphorylation. Numerous studies have been conducted regarding histone modifications in WAT, which have been extensively reviewed elsewhere (49, 50, 51). In the present review, we briefly outline the roles of histone modifications in regulating adipogenesis and adipocyte function. During adipogenesis, histone-modifying enzymes promote dramatic and dynamic chromatin remodeling and drive the formation of active chromatin regions that are enriched with crucial *cis*-regulatory elements, adipogenic genes, or both (52). Such chromatin remodeling facilitates the cooperative binding of multiple TFs, including retinoid X receptor, C/EBPs, and PPARγ, thus initiating the expression of early adipogenic genes (52). In mature adipocytes, lysine-specific demethylase-1 (LSD1) has been proposed to regulate the expression of metabolic genes in adipocytes (53). The inhibition of LSD1 activity in differentiating adipocytes *in vitro* provokes a significant increase in mono-methylated H3K4 (H3K4me), upregulating the expression of genes associated with lipid metabolism (*e.g.*, PPARγ coactivator-1a [PGC1α], adipose triglyceride lipase, and fatty acid transporter protein 1 [FATP1]). In obesity, adipose LSD1 expression is aberrantly upregulated, leading to impeded expression of PGC1α and FATP1, contributing to WAT dysfunction (53).

### DNA methylation in transcriptional regulation

**Figure 2:**
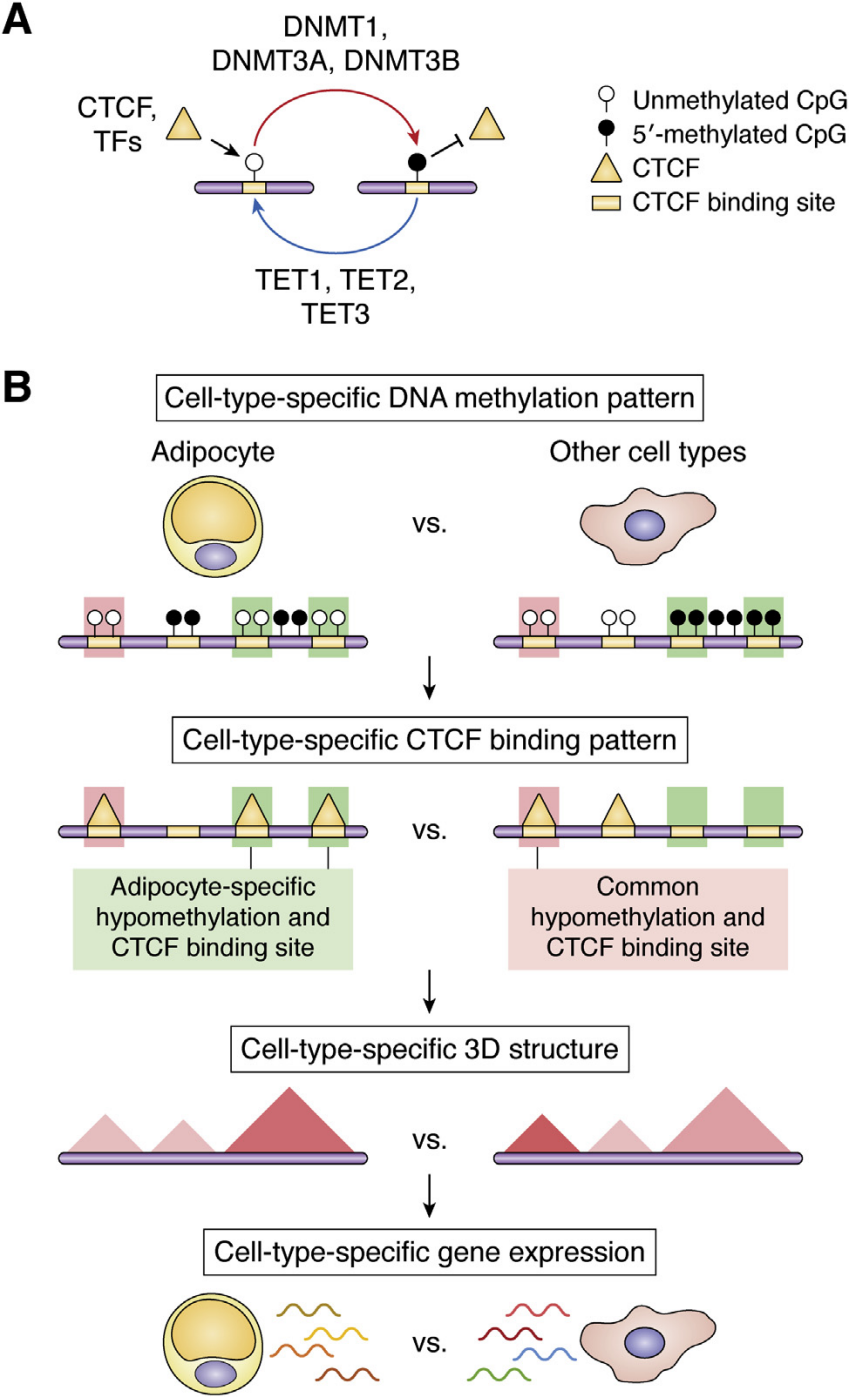
**Regulation of cell-type-specific gene expression by the differential pattern of DNA methylation.***A*, interaction between methylated DNA and CCCTC-binding factor (CTCF) or transcription factors (TFs). DNA methylation and demethylation can be mediated *via* DNMTs and TETs, respectively. Unmethylated CpG region can interact with CTCF or TFs, whereas methylated CpG region is associated with suppressed CTCF binding, which is associated with an altered chromosomal structure. *B*, distinct DNA methylation patterns of adipocytes can be linked to adipocyte-specific CTCF-binding patterns, modulating adipocyte-specific gene expression by forming certain 3D structures.

DNA methylation is the most common DNA modification, which preferentially occurs at a cytosine immediately 5′ to guanine (CpG sites) and, to a lesser extent, in a non-CpG context (54). Adding a methyl group to cytosine is catalyzed by DNA methyltransferases (DNMTs), including DNMT1, DNMT3A, and DNMT3B. DNMTs use SAM as the methyl donor. In contrast, DNA demethylation can be achieved in two ways (https://www.who.int/news-room/fact-sheets/detail/obesity-and-overweight): passive demethylation owing to the lower level of DNMT activity or paucity of SAM availability and (1) active demethylation by ten-eleven translocation (TET) enzymes that promote the sequential oxidation of methyl groups followed by DNA repair processes (Fig. 2*A*). Both DNMTs and TETs sensitively respond to external (*e.g.*, diet), internal (*e.g.*, hormones), and genetic factors, directing gene expression and maintaining or altering genomic architecture. In particular, the DNA methylation pattern set up by the cooperation between DNMTs and TETs is a crucial regulatory mechanism for maintaining cell-type-specific gene regulation (55).

DNA methylation, particularly in the regulatory elements within or near core promoter regions, is associated with transcription repression, either directly (by blocking the accessibility of TFs) or indirectly (by recruiting other repressive proteins with methyl-binding domains, including MeCP2 and MBD proteins). In particular, the latter mechanism may contribute to the stable repression of thousands of genes involved in various biological pathways, including development and proliferation (56). However, recent progress in the method (*e.g.*, Hi-C and ChIA-PET) that enables the mapping of regulatory interactions from genome-wide epigenetic datasets challenged the conventional idea that DNA methylation at proximal regulatory elements (*e.g.*, promoter) primarily contributes to the biological functions of DNA methylation (57, 58). The findings obtained using new techniques suggest that in addition to activity control of *cis*-regulatory elements, DNA methylation could have broader regulatory effects on transcription through the regulation of long-range genomic interactions formed by 3D chromosome conformation (Fig. 2*B*). In addition, as opposed to DNA methylation in proximal regulatory elements, DNA methylation involved in long-range genomic interactions does not comply with a linear mode of direct repression of transcription. Rather, DNA methylation can have differential effects on regional gene expression according to the context-dependent properties of DNA methylation sites (*e.g.*, TAD boundary-associated sites and intra/inter TAD interaction sites) in the formation of functional chromosomal looping (27, 59).

### Classic and newly discovered roles of DNA methylation in WAT and obesity

Global and locus-specific differential DNA methylation has been reported in humans with obesity and rodent models of obesity. Several clinical studies have identified various genes such as *FTO*, *IRS1*, and *HIF3A*, whose DNA methylation and gene expression in adipocytes are associated with obesity-induced WAT expansion and type 2 diabetes (35, 37, 60). Furthermore, an epigenome-wide association study with 5387 individuals identifies 187 CpGs sites linked to adiposity (61). These 187 CpGs sites are strongly enriched in active chromatin sites such as DNase hypersensitivity sites and the activating histone marks, H3K4me1 and H3K27ac, in WAT, suggesting the potential regulatory roles of differentially methylated CpGs in the expression of adiposity-associated genes. More importantly, 187 CpGs sites are located within 500 kbp of genes, with many having established roles in adipose tissue biology and insulin resistance (*e.g.*, *ABCG1*, *LPIN1*, *HOXA5*, *LMNA*, *CPT1A*, *SOCS3*, *SREBF1*, *and PHGDH*). Likewise, obesity induces widespread alterations in DNA methylation in the adipocytes of diet-induced obese mouse models (62). In that study, 232 differentially methylated regions (DMRs) correlated with the obesity status, and these DMRs were near-genes that were significantly overrepresented in lipid metabolism and immune/inflammatory pathways (*e.g.*, Tcf7l2, Pck, Fbxw8, and Akt2). In addition to association studies using genetically engineered knockout mouse models, several studies have shown the causal roles of DNA methylation modifiers in WAT dysfunction by regulating DNA methylation in near-core promoter regions of key adipokines, including adiponectin and FGF21 (40, 42). Although these studies have demonstrated the close functional association of DNA methylation with WAT biology, they have focused on the regulatory nature of differentially methylated CpGs in the promoter regions of genes or, if not, the correlation of differential methylation in CpGs with their nearby genes (*e.g.*, located within 500 kbp from the CpGs).

**Figure 3:**
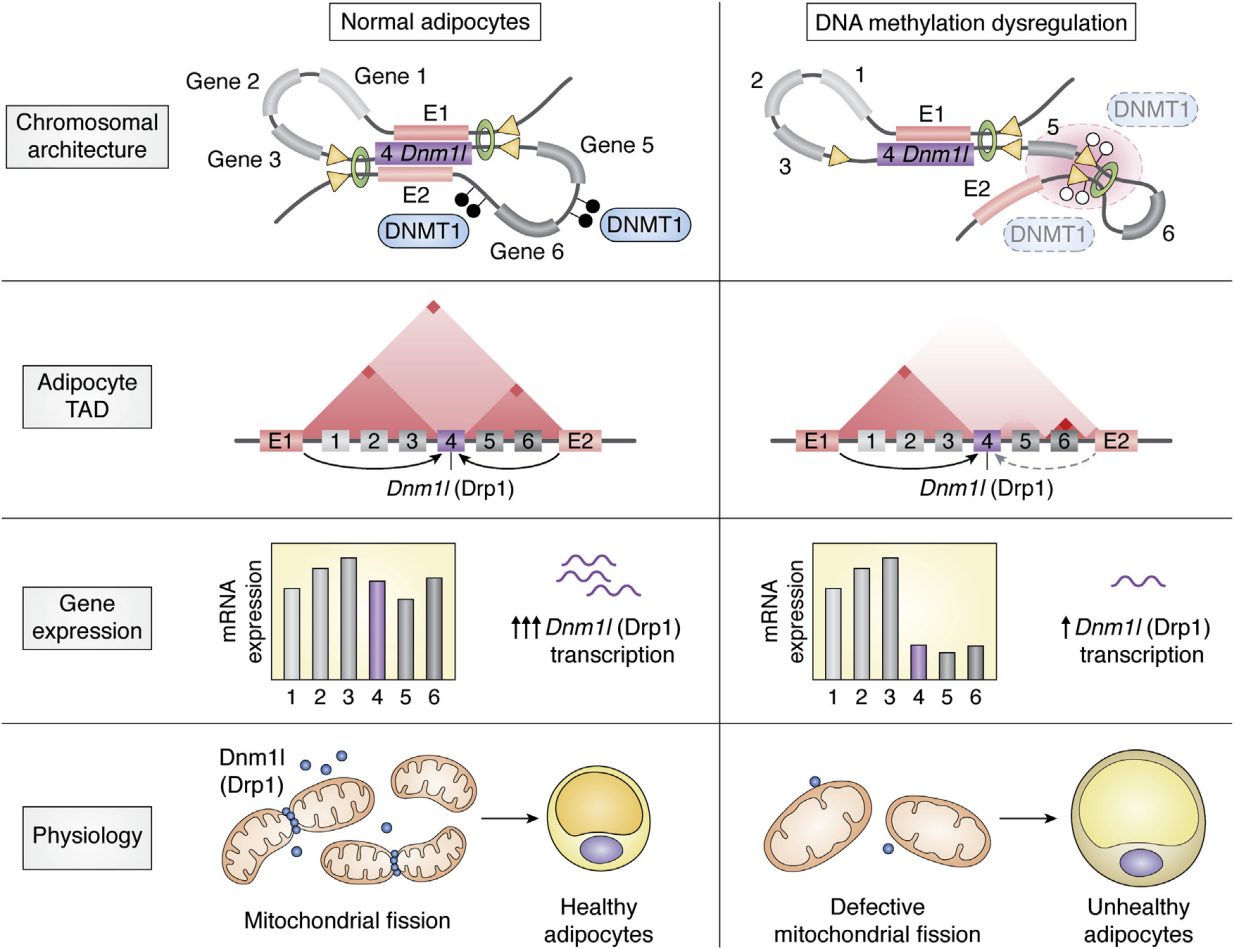
**Role of DNA methylation in maintaining CTCF-mediated chromosome architecture in adipocytes.** In adipocytes, the expression of many genes is regulated by synergistic action of multiple *cis*-regulatory elements (*e.g.*, enhancers), often remote from target genes. Physical interactions of distal enhancers and their target promoter are facilitated through the formation of chromosomal loops mediated by adipocyte-specific CTCF bindings. Such adipocyte-specific CTCF-binding landscape is retained by DNA methylation that suppresses aberrant CTCF binding along the genome. The loss of DNA methylation by DNMT1 ablation leads to aberrant CTCF binding, which abrogates long-range genomic interactions in expressing several genes (Gene 4–6) in specific TADs, which include Gene 4 (Dnm1l)—the coding gene of Drp1. Because Drp1 is crucial for efficient mitochondrial fission, DNMT1 ablation decreases mitochondrial bioenergetics in white adipocytes. Such deleterious changes in white adipocytes through Dnmt1 depletion provoke defective mitochondrial homeostasis, ultimately leading to WAT dysfunction.

However, the functions of a large number of differentially methylated CpGs located within the intergenic and likely regulatory region have been barely established because identifying target genes and subsequent biological pathways of these CpGs has been challenging in human obesity and associated metabolic traits. One issue related to this is that assigning noncoding regulatory elements to the target gene(s) is not immediately evident in the systemic mapping of DNA methylation, thus hindering the understanding of biological mechanisms through which these DNA methylation loci contribute to WAT function and obesity. In addition, the potential role of DNA methylation in maintaining 3D genomic structure in adipocytes has remained unclear. To address this knowledge gap, we have recently integrated multilayer genomic data, including pCHi-C, ChiP-seq, bisulfite sequencing data, and RNA sequencing data (63). Our comparative epigenomic analysis demonstrates that adipocyte-specific DNA methylation pattern was engaged in the activity control of distal enhancers whose target genes are closely linked to adipocyte biology (Fig. 2*B*). Furthermore, DNA methylation is closely associated with establishing the binding landscape of the key architectural protein, CTCF, which consolidates the 3D genomic structure required for chromosomal looping in adipocytes. Approximately 50% of adipocyte-specific CTCF binding coincides with the DNA methylation loci specifically hypomethylated in adipocytes compared with the liver. In contrast, only 9.2% of constitutive CTCF-binding sites occupied in all analyzed mouse cell types (29 mouse cell types) show differential methylation in adipocytes. These observations highlight a close association of DNA methylation with the adipocyte-specific CTCF-binding landscape and consequent consolidation of chromosomal looping in adipocytes. Notably, the expression of several adipocyte genes is modulated through the converging effects of DNA methylation on multiple distal enhancers (three enhancers/genes) and their interactions with target genes. For instance, the expression of crucial adipose genes such as PPARγ, KLF5, and CD36 entails concerted action of enhancer hypomethylation and strong long-range genomic interactions. On the other hand, the collaboration of intensive long-range genomic interactions with enhancer hypermethylation significantly suppresses genes (*e.g.*, ACAT3, APOA4, and APOC3) whose expression is significantly repressed in adipocytes. We further reveal that the loss of DNA methylation through the ablation of adipocyte DNMT1, the most abundant DNA methylation modifier in WAT, leads to aberrant CTCF binding, where DNA methylation is decreased by DNMT1 depletion. Such changes in CTCF-binding patterns are associated with the destabilization of intra-TAD interactions, leading to dysregulated transcription in adipocytes (Fig. 3). In particular, the expression of Drp1 (encoded by *Dnm1l*), a key regulator of mitochondrial fission, is significantly reduced by impaired promoter–distal enhancer interactions mediated by adipocyte DNMT1 depletion (63). Decreased Drp1 expression abrogates mitochondrial bioenergetics by inhibiting mitochondrial fission and promotes aberrant lipid metabolism in adipocytes, rendering adipocyte hypertrophy, impaired adipocyte progenitor proliferation, and WAT dysfunction. These findings provide proof of concept that DNA methylation can render WAT function through the simultaneous modulation of *cis*-regulatory element activity and chromosome architecture.

## Conclusions

The incidence of obesity and metabolic diseases has risen rapidly during the past few decades. Epigenetic modification is considered a key mechanism linking obesity and metabolic diseases. In addition to the function of DNA methylation in *cis*-regulatory element activity control, there is now new evidence suggesting that DNA methylation can also function as the crucial regulatory mechanism that maintains intact chromosome loops for the expression of genes associated with WAT function. Given that defective chromosome loops within TADs have multiple impacts on genes, these new findings implicate that altered DNA methylation in even a small number of loci has relatively huge impacts on transcription regulation. Furthermore, because different fat depots, including visceral WAT, subcutaneous WAT, and brown adipose tissue, are equipped with distinct DNA methylation landscapes, investigating whether DNA methylation mediates fat depot-specific function by maintaining distinct chromosome loops in different fat depots will be interesting. In addition, nutritional status during prenatal or early life has been suggested to contribute to an aberrant DNA methylation landscape, which profoundly affects the risks of metabolic disorders later in life (64, 65, 66). Thus, the role of DNA methylation in the regulation of 3D chromosome architecture may provide new insights into understanding the mechanistic connection of dysregulated WAT-specific DNA methylome in early life with metabolic pathologies observed in later life (67). Thus, determining regulatory mechanisms and identifying biological functions of DNA methylation in WAT will provide a new approach to combat obesity and metabolic diseases.

## Conflict of interest

The authors declare that they have no conflicts of interest with the contents of this article.
